# Pulmonary arterial distensibility - 2D phase contrast vs 2D bSSFP

**DOI:** 10.1186/1532-429X-14-S1-P85

**Published:** 2012-02-01

**Authors:** Alejandro Roldán-Alzate, Oliver Wieben, Alex Frydrychowicz, Naomi C Chesler, Christopher Francois

**Affiliations:** 1 Radiology, University of Wisconsin, Madison, WI, USA; 2 Medical Physics, University of Wisconsin, Madison, WI, USA; 3 Biomedical Engineering, University of Wisconsin, Madison, WI, USA

## Summary

Main pulmonary artery distensibility is a strong predictor of mortality in patients with pulmonary arterial hypertension. This study shows that either 2D PC or 2D bSSFP can be used to reliably non-invasively assess it by measuring MPA relative area change.

## Background

The pulmonary circulation is a highly compliant system that generates little resistance to blood flow. However, in the presence of pulmonary arterial hypertension (PAH), blood pressure and vascular resistance in the pulmonary circulation are elevated. This leads to distension and stiffening of the main pulmonary artery (MPA) and vessel wall remodeling, which in itself may influence stiffness [1]. Proximal arterial distensibility is a parameter that depends mainly on the anatomy (geometry) of the vessel and can be estimated noninvasively with cross-sectional imaging techniques. In particular, the relative area change (RAC) of the MPA, which is inversely proportional to arterial stiffness, is a strong predictor of mortality in patients with PAH [2]. The purpose of this study was to compare two non-invasive magnetic resonance imaging (MRI) methods for quantification of MPA distensibility using an acute PAH dog model.

## Methods

Six adult female beagles were anesthetized with isoflurane. MRI was performed prior to and following injection of micro-beads into the right atrium and ventricle to induce PAH, resulting 12 comparison studies. The presence of PAH was confirmed by right heart catheterization (RHC). All MR images were acquired on a 3T scanner (MR750, GE Healthcare, Waukesha, WI). Double-oblique images perpendicular to the direction of the flow in the MPA were obtained using ECG-triggered 2D CINE balanced steady-state free precession (bSSFP) and through-plane velocity-encoded 2D phase contrast (PC) at the same level [3]. PC and bSSFP images were segmented using dedicated cardiovascular analysis software (CV-Flow and and MASS-Analysis, respectively, Medis, Leiden, NL). Maximum and minimum MPA areas (A_max_ and A_min_, respectively) were used to calculate RAC = (A_max_-A_min_)/A_max_. Bland-Altman analysis was used to study the differences between PC and bSSFP to calculate A_max_, A_min_, and RAC. Student t-test was used to evaluate statistical significance of the differences between techniques in all three paramenters.

## Results

The mean values for RAC, A_max_ and A_min_ were 36.38±7.86%, 241.98±71.62cm^2^, and 157.06±58.51cm^2^ for PC, respectively and 31.14±6.95%, 220.88±62.76cm^2^, and 155.34±58.38cm^2^ for bSSFP, respectively (p=0.10, 0.45, and 0.94, respectively). The mean differences for RAC, A_max_ and A_min_ were -5.23±8.44%, -21.10±40.67cm^2^, -1.72±40.89cm^2^, respectively.

## Conclusions

No statistically significant difference is present between PC and bSSFP for measuring RAC, A_max_ or A_min_. The differences between the two methods for measuring these parameters are small suggesting that either technique (PC or bSSFP) can be used to reliably non-invasively measure MPA distensibility. A benefit of using PC for measuring RAC is that it can also be used to quantify blood flow.

## Funding

University of Wisconsin - Madison, Department of Radiology, Research and Development fund.

**Figure 1:**
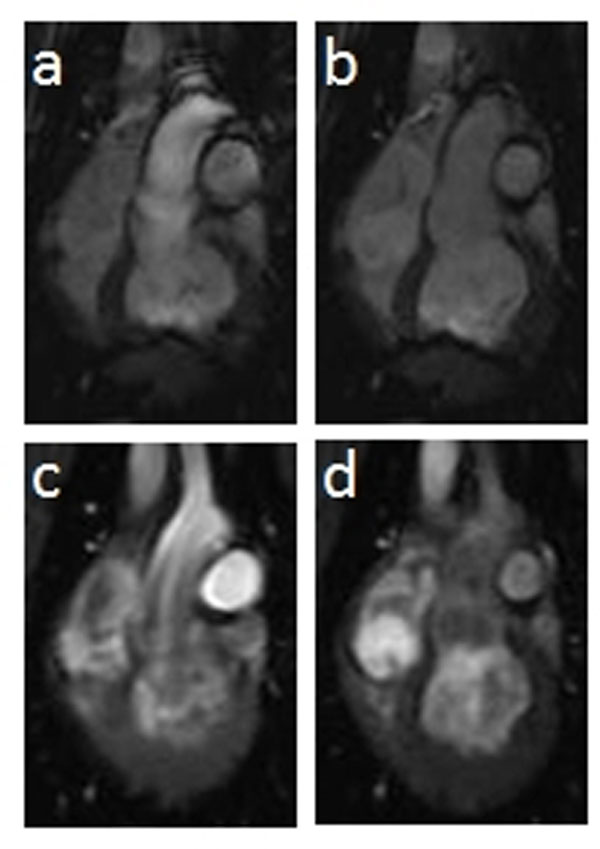
2D bSSFP images (a. A_max_ and b. A_min_) and 2DPC images (c.A_max_ and d. A_min_).

**Figure 2:**
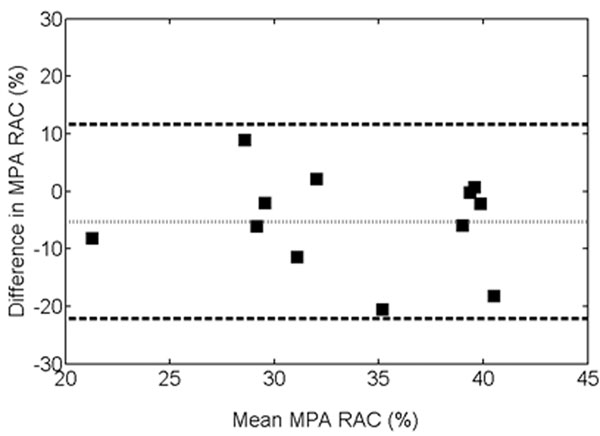
Bland-Altman plot for comparison of MPA RAC measured with 2D PC and 2D bSSFP.
